# Effects of alpha-mangostin on the expression of anti-inflammatory genes in U937 cells

**DOI:** 10.1186/1749-8546-7-19

**Published:** 2012-08-24

**Authors:** Szu-Hsiu Liu, Lain-Tze Lee, Nai-Yun Hu, Kuo-Kuei Huange, Ying-Chu Shih, Iinuma Munekazu, Jen-Ming Li, Ting-Yu Chou, Wei-Hsin Wang, Ting-Shou Chen

**Affiliations:** 1 Herbal Medicinal Product Technology Division Pharmacognosy Laboratory, Industrial Technology Research Institute, Hsinchu, 30011, Taiwan; 2 Laboratory of Pharmacognosy, Gifu Pharmaceutical University, Gifu, 501-1196, Japan; 3 Strategic Business & Innovation Technology Development Division, Industrial Technology Research Institute, Hsinchu, 30011, Taiwan; 4 In Vitro Diagnostics Division, Industrial Technology Research Institute, Hsinchu, 30011, Taiwan; 5 Industrial Technology Research Institute, 321 Sec.2, Kuang Fu Road, Hsinchu, 30011, Taiwan

## Abstract

**Background:**

α-Mangostin (α-MG) is a main constituent of the fruit hull of the mangosteen. Previous studies have shown that α-MG has pharmacological activities such as antioxidant, antitumor, anti-inflammatory, antiallergic, antibacterial, antifungal and antiviral effects. This study aims to investigate the anti-inflammatory molecular action of α-MG on gene expression profiles.

**Methods:**

U937 and EL4 cells were treated with different concentrations of α-MG in the presence of 0.1 ng/mL lipopolysaccharide (LPS) for 4 h. The anti-inflammatory effects of α-MG were measured by the levels of tumor necrosis factor (TNF)-α and interleukin (IL)-4 in cell culture media, which were determined with enzyme-linked immunosorbent assay kits. The gene expression profiles of all samples were analyzed with a whole human genome microarray, Illumina BeadChip WG-6 version 3, containing 48804 probes. The protein levels were determined by Western blotting analyses.

**Results:**

α-MG decreased the LPS induction of the inflammatory cytokines TNF-α (*P* = 0.038) and IL-4 (*P* = 0.04). α-MG decreased the gene expressions in oncostatin M signaling *via* mitogen-activated protein kinase (MAPK) pathways, including extracellular signal-regulated kinases (*P* = 0.016), c-Jun N-terminal kinase (*P* = 0.01) , and p38 (*P* = 0.008). α-MG treatment of U937 cells reduced the phosphorylation of MAPK kinase 3 / MAPK kinase 6 (*P* = 0.0441), MAPK-activated protein kinase-2 (*P* = 0.0453), signal transducers and activators of transcription-1 (STAT1) (*P* = 0.0012), c-Fos (*P* = 0.04), c-Jun (*P* = 0.019) and Ets-like molecule 1 (Elk-1) (*P* = 0.038).

**Conclusion:**

This study demonstrates that α-MG attenuates LPS-mediated activation of MAPK, STAT1, c-Fos, c-Jun and EIK-1, inhibiting TNF-α and IL-4 production in U937 cells.

## Background

The mangosteen fruit has been used in Chinese and Ayurvedic medicine [1]. Extracts of mangosteen have antioxidant, antitumor, anti-inflammatory, antiallergic, antibacterial, antifungal and antiviral effects [1-3]. α-Mangostin (α-MG), which was first isolated from the mangosteen in 1855, is a competitive antagonist of the histamine H_1_ receptor and possesses many biological properties, such as anti-inflammatory, anti-oxidative damage and antioxidant activities [4-6]. Previous studies have shown that α-MG significantly inhibits nitric oxide (NO), prostaglandin E2 (PGE2), tumor necrosis factor (TNF)-α and inducible NOS (iNOS) production in lipopolysaccharide (LPS)-stimulated RAW 264.7 cells [3,6,7]. PGE2, TNF-α and iNOS are cytokines involved in inflammatory processes, including increased vascular permeability, vascular dilation and neutrophil chemotaxis [8,9].

LPS stimulation of human monocytes activates several intracellular signaling pathways that include the IκB kinase (IKK) and nuclear factor-κB (NF-κB) pathway and three mitogen-activated protein kinase (MAPK) pathways: extracellular signal-regulated kinases 1 and 2 (ERK1/2), c-Jun N-terminal kinase (JNK) and p38 [10]. In turn, these signaling pathways activate a variety of transcription factors including NF-κB (p50/p65) and activator protein 1 (AP-1; c-Fos/c-Jun), which coordinate the induction of many genes encoding inflammatory mediators [10]. However, the anti-inflammatory molecular effects of the α-MG action remains unclear. Exposure of U937 cells, a human myeloid leukemic cell line, to different concentrations of α-MG allows examination of the transcriptional responses and investigation of the exact intracellular effects of α-MG using complementary DNA (cDNA) microarrays.

This study aims to investigate the effects of the α-MG on the expression of three MAPK pathways, ERK1/2, JNK and p38 in cultured U937 cells.

## Methods

### Chemicals and reagents

LPS (from *Escherichia coli*), RPMI 1640 medium, 3-(4,5-dimethyl-2-thiazolyl)-2,5-diphenyl-2 H-tetrazolium bromide (MTT), phosphate-buffered saline (PBS), antibiotics, L-glutamine and trypsin-EDTA were purchased from Gibco BRL (USA). Fetal bovine serum was purchased from Hyclone Laboratories Inc. (USA). Enzyme-linked immunosorbent assay (ELISA) test kits for Human IL-4 DuoSet and Human TNF-α DuoSet were obtained from R&D Systems (USA). Phospho-p38 MAPK (Thr180/Tyr182) rabbit monoclonal, phospho-SAPK/JNK (Thr183/Tyr185) rabbit monoclonal, phospho-ERK1/2 (Thr202/Tyr204) rabbit monoclonal, phospho- EIK-1 (Ser383) rabbit monoclonal, phospho-c-Fos (Ser32) rabbit monoclonal, phospho-c-Jun (Ser63) rabbit monoclonal, phospho-MMK3/MMK6 (Ser189/207) rabbit monoclonal, phospho-MAPKAPK-2 (Thr334) rabbit monoclonal, phospho-STAT1 (Try701) rabbit monoclonal, c-Fos rabbit monoclonal and c-Jun rabbit monoclonal antibodies were obtained from Cell Signaling Technology (USA). α-MG was provided by Dr. Iinuma Munekazu. A Bradford assay kit was purchased from Bio-Rad (Germany). A mirVana miRNA Isolation Kit was purchased from Ambion Inc. (USA). All other chemicals were purchased from Sigma-Aldrich (USA).

### Cell culture

The human myeloid leukemic cell line U937 (ATCC No. CRL-1593.2) was purchased from the American Type Culture Collection (USA). The cells were cultured in RPMI 1640 medium supplemented with 10% (v/v) fetal bovine serum, 2 mM glutamine, 100 U/mL penicillin and 100 μg/mL streptomycin at 37°C under 5% CO_2_. EL4 cells were purchased from the American Type Culture Collection (ATCC TIB-39). The cells were cultured in Dulbecco’s modified Eagle’s medium supplemented with 10% (v/v) fetal bovine serum, 2 mM glutamine, 100 U/mL penicillin and 100 μg/mL streptomycin at 37°C under 5% CO_2_.

### Differentiation induction

U937 cells were cultured in RPMI 1640 medium supplemented with 10% (v/v) fetal calf serum, 2 mM glutamine, 100 U/mL penicillin and 100 μg/mL streptomycin at 37°C under 5% CO_2_. For differentiation induction, the cells were seeded at a density of 2 × l0^7^ cells per T150 flask. Differentiation was initiated by addition of phorbol-12-myristate-13-acetate to the culture medium to a final concentration of 50 ng/mL and allowed to proceed for 24 h. The U937 cells were then washed with complete culture media once and incubated in U937 culture medium for 48 h.

### TNF-α and IL-4 cytokine assays

Differentiated U937 and EL-4 cells were aliquoted into the wells of 96-well plates at a density of 1.6 × 10^5^ cells/well, mixed with different concentrations of α-MG. U937 cells were treated with 7.6, 12.5, 30.5 nM, respectively. EL-4 cells were treated with 3.0, 6.1, 12.2 nM, respectively and incubated for 30 min at 37°C under 5% CO_2_. LPS (0.1 ng/mL) was added to the differentiated U937 and EL-4 cells and incubated for 4 h. The culture media were then harvested for TNF-α analyses with Human TNF-α DuoSet ELISA kit (R&D Systems, USA). The culture media were harvested for IL-4 analyses with Human IL-4 DuoSet ELISA kit (R&D Systems, USA). The half maximal inhibitory concentration (IC_50_; μg/mL) was calculated with GraFit software (version 7; GraFit Data Analysis Software, USA).

### Cytotoxicity assay

Cytotoxicity assays were performed by the MTT method. Cells were incubated with 100 μL of 1 mg/mL MTT for 1 h at 37°C under 5% CO_2_. DMSO (100 μL) was added to dissolve the crystals and the OD_560_ was measured with an ELISA reader (Spectrafluor Plus, Tecan, Switzerland). The results were expressed as cell viability percentages among LPS-stimulated cells.

### Microarray analysis

#### RNA isolation

U937 cells were harvested into pellets, washed with PBS and stored at −80°C until extraction. Total RNA and small RNAs from the cultured cells were isolated using the mirVana miRNA Isolation Kit.

### Gene expression

The gene expression profiles were analyzed using a whole human genome microarray containing 48,804 probes (BeadChip WG-6 version 3; Illumina Inc., USA). Biotin-labeled complementary RNA (cRNA) for hybridization was generated by *in vitro* transcription based on the Eberwine protocol using Illumina Human Whole Genome-6 expression BeadChip kits (Illumina Inc, USA). Total RNA (500 ng) was reverse-transcribed into cDNA, followed by linear amplification steps according to an Illumina TotalPrep RNA Amplification Kit (Ambion Inc., USA). Hybridization was performed with 1.5 μg of biotin-labeled cRNA in each BeadChip WG-6 array. After incubation at 58°C for 16 h, the BeadChip WG-6 was washed with fresh wash tray according to Illumina Whohle-Genome Gene Expression Direct Hybridization Assay, stained with streptavidin-Cy3 dye (Amersham Biosciences, Buckinghamshire, UK) and scanned as described in the Illumina manual. The HumanWG-6 v3.0 Expression BeadChip WG-6 contains six arrays on a single BeadChip WG-6, each with 48,804 probes derived from human genes in the NCBI RefSeq and UniGene databases. Each array on the BeadChip WG-6 covers genome-wide transcription of well-characterized genes, gene candidates and splice variants. The intensity of each probe was calculated as the average intensity of at least 15 beads. Array images and data output were processed using Illumina BeadStudio software (Ambion Inc, USA). The analysis methods for the gene expressions using R and BioConductor 2.10 Software Packages (Biobase, beadarray, limma packages of R/BioConductor were used).

### Gene expression profiling

The gene expression profiles of undifferentiated and differentiated U937 cells were determined using the Illumina WG-6 version 3 Beadarray (Illumina Inc., USA). The raw intensity of spots was log-2 transformed for subsequent analysis. Quantile normalization was performed within all arrays to adjust the systematic variation of experiments and dye effects. Significantly changed genes were identified by Limma test with BH (Benjamini & Hochberg) adjust *P* values of less than 0.05.

### Pathway and gene ontology analysis

The pathway and gene ontology analyses were performed using the MetaCore software (GeneGo Inc., USA), in which the differentially expressed gene sets for LPS and α-MG comprised the significantly changed genes between the two conditions and were annotated according to their biological processes based on gene ontology information.

### Western blot analysis

Differentiated U937 cells at a density of 4 × 10^6^ cells/well were pretreated with 13.4 nM α-MG for 30 min. The U937 culture medium contained 0.1 ng/mL LPS, and the incubation was continued for 4 h at 37°C under 5% CO_2_. The cells were washed twice with ice-cold PBS, resuspended in lysis buffer (20 mM Tris–HCl pH 7.5, 150 mM NaCl, 1 mM EDTA, 1 mM EGTA, 1% Triton X-100, 2.5 mM sodium pyrophosphate, 1 mM β-glycerophosphate, 1 mM Na_3_VO_4_, 1 μg/mL leupeptin, 1 mM PMSF) and centrifuged (Thermo Fisher Scientific Laboratory, USA) at 16,000 × *g* for 15 min at 4°C. The clarified cell lysates were used for Western blot analyses. The protein concentrations were determined using the Bradford assay kit (Ambion Inc., USA).

Protein extracts (20 μg) under reduced conditions were fractionated by 10% sodium dodecyl sulfate-polyacrylamide gel electrophoresis and transferred to Hybond nitrocellulose membranes. The membranes were blocked with 3% non-fat milk in Tris-buffered saline containing 0.1% Tween for 1 h. The activations of p38, MAPK, JNK, ERK1/2, EIK-1, c-Fos, c-Jun, MAPK kinase 3 / MAPK kinase 6 (MMK3/MMK6), MAPK-activated protein kinase-2 (MAPKAPK-2) and signal transducers and activators of transcription-1 (STAT1) were assessed using phospho-p38 MAPK (Thr180/Tyr182) rabbit monoclonal, phospho-SAPK/JNK (Thr183/Tyr185) rabbit monoclonal, phospho-ERK1/2 (Thr202/Tyr204) rabbit monoclonal, phospho-EIK-1 (Ser383) rabbit monoclonal, phospho-c-Fos (Ser32) rabbit monoclonal, phospho-c-Jun (Ser63) rabbit monoclonal, phospho-MMK3/MMK6 (Ser189/207) rabbit monoclonal, phospho-MAPKAPK-2 (Thr334) rabbit monoclonal, phospho-STAT1 (Try701) rabbit monoclonal, c-Fos rabbit monoclonal and c-Jun rabbit monoclonal antibodies according to the manufacturer’s instructions. The antibody-bound protein bands were visualized by incubation with a horseradish peroxidase-conjugated secondary antibody (Sigma-Aldrich, USA), followed by detection using the ECL system (Amersham Pharmacia Biotech, USA). The integrated optical densities of the bands were quantified using Image J software (NIH, USA). Each sample was normalized by the β-tubulin content, as a constitutively expressed protein.

### Statistical analysis

All experiments were performed in triplicate and repeated independently at least three times. Data were presented as mean ± standard deviation (SD) and analyzed by one-way analysis of variance (ANOVA) using SAS 9.1.3 software (SAS Institute Inc., USA) followed by a Tukey test to determine any significant differences. *P* values less than 0.05 were considered statistically significant. Dose dependence was visually determined from the dose–response graphs.

## Results and discussion

### Inhibition of LPS-induced TNF-α and IL-4 production

**Figure 1:**
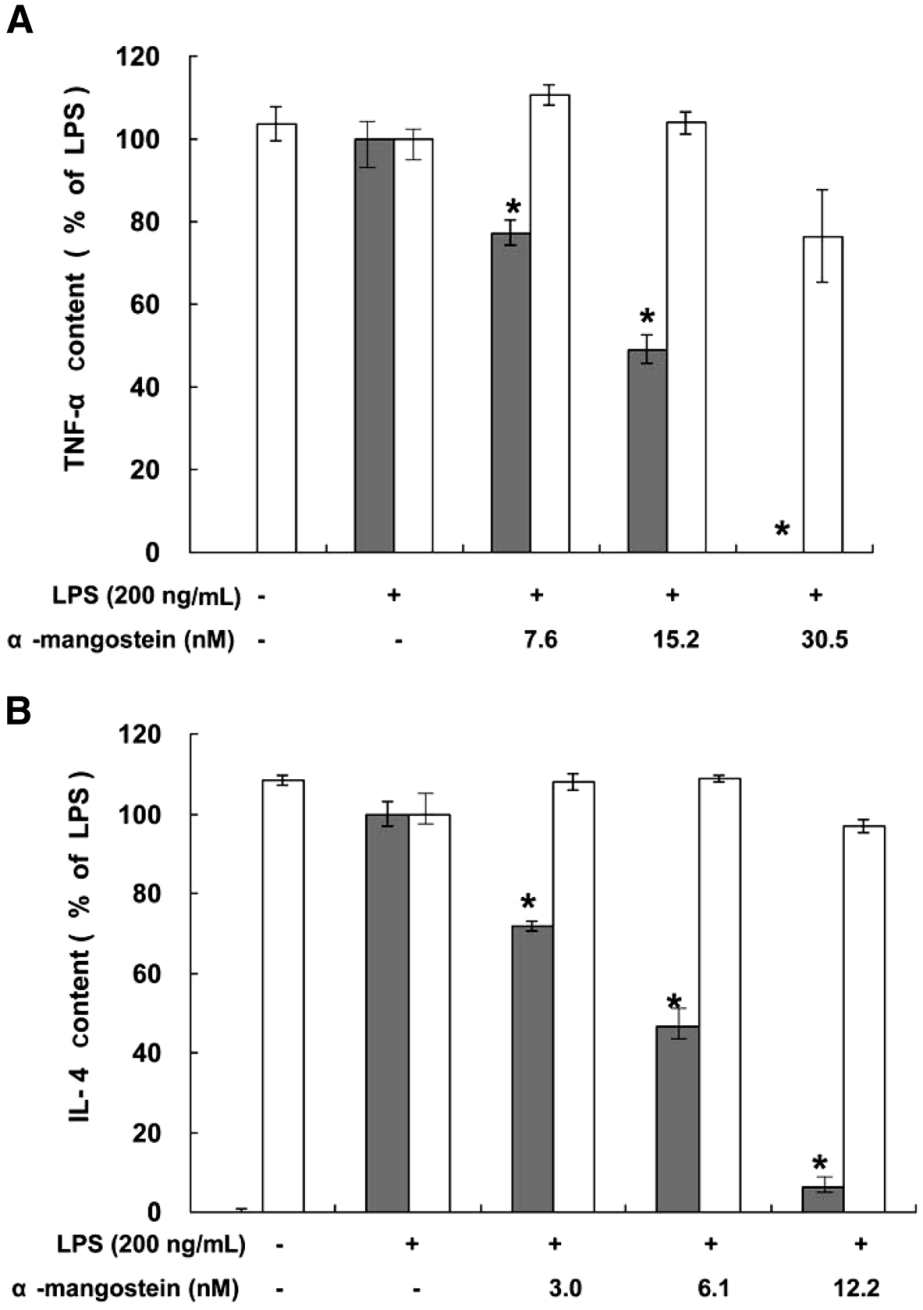
**Inhibition of TNF-α and IL-4 secretion from U937 and EL4 cells by α-MG.** (**A**) U937 cells were treated with 0.1 ng/mL LPS in the presence or absence of different concentrations of α-MG, 7.6, 12.5, 30.5 nM, respectively for 4 h. TNF-α secreted into the conditioned media was quantified by ELISA. The TNF-α content (gray bars) and cell viabilities (open bars) are shown. (**B**) EL4 cells were treated with 0.1 ng/mL LPS in the presence or absence of different concentrations of α-MG, 3.0, 6.1, 12.2 nM, respectively for 18 h. IL-4 secreted into the conditioned media was quantified by ELISA. The IL-4 content (gray bars) and cell viabilities (open bars) are shown. All experiments were performed in triplicate and repeated independently three times. *P < 0.05, significant difference from LPS treatment.

LPS significantly induced the production of TNF-α and IL-4 in U937 cells. The inhibitory effects of α-MG on inflammatory cytokines were evaluated by measuring the amounts of secreted TNF-α and IL-4 in LPS-stimulated U937 cells after treatment with α-MG. α-MG inhibited the production of TNF-α (*P* = 0.038) (Figure 1A) and IL-4 (*P* = 0.04) (Figure 1B) in a dose-dependent manner. The anti-inflammatory effects of α-MG could be attributed to the inhibition of inflammatory cytokine production or a reduction in the number of U937 cells through cytotoxicity. The latter possibility was excluded by comparing the numbers of cells cultured with the different concentrations of α-MG, wherein no significant decreases in cell viability were observed when the concentration was below 15.2 nM (*P* = 0.1) (Figure 1A). The IC_50_ of α-MG was 13.4 ± 0.4 nM.

### Microarray analysis

**Figure 2:**
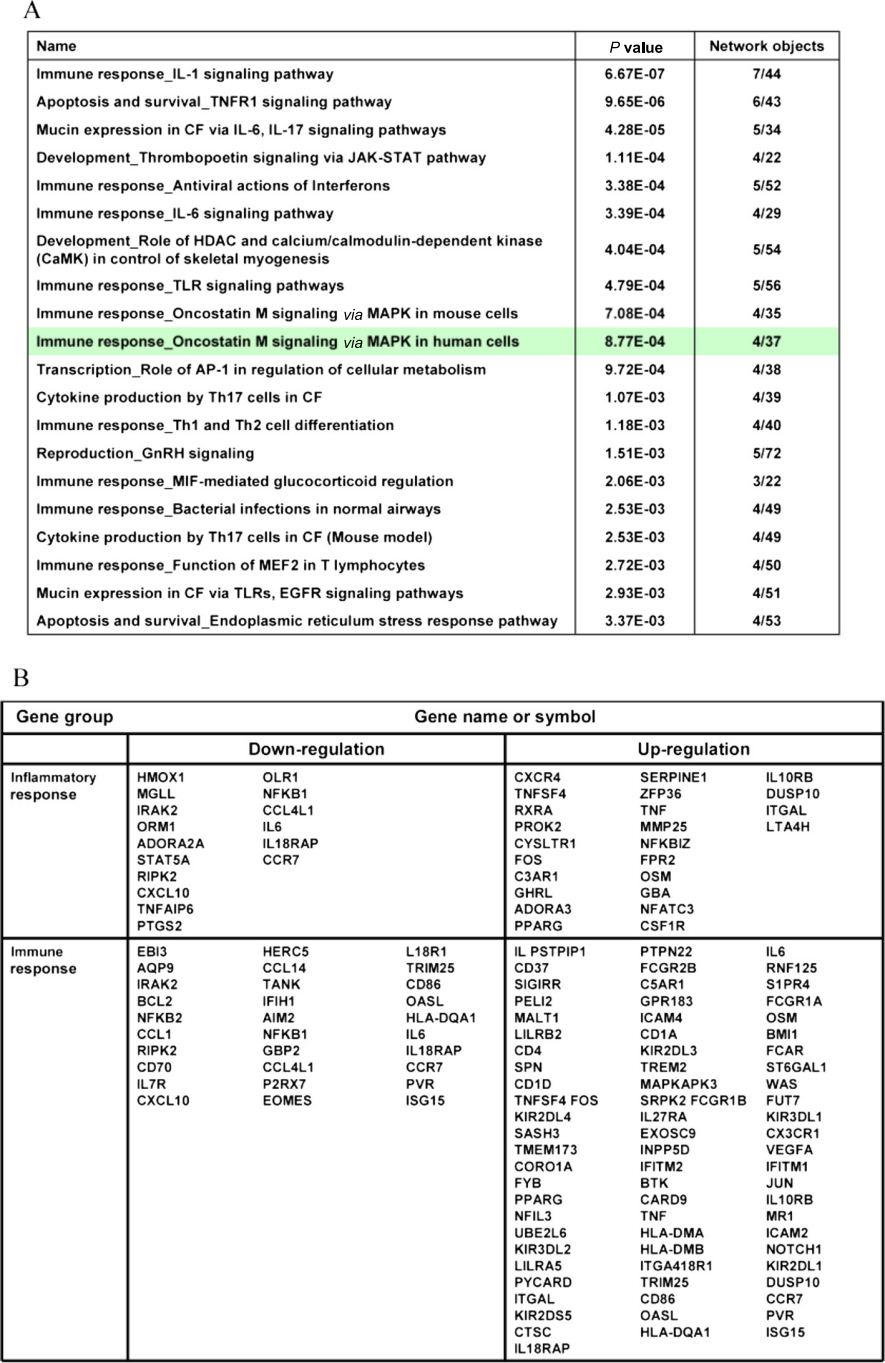
**Microarray analysis of α-MG in LPS-stimulated U937 cells.** (**A**) Analysis of α-MG-regulated pathways using the MetaCore database (most significant 20 pathways). (**B**) Groups of genes suppressed or induced following exposure to α-MG.

Treatment of LPS-stimulated U937 cells with 13.4 nM α-MG changed the gene expression pattern (Figure 2A). There were 1536 and 1491 significantly changed genes at 1 and 6 h with LPS and the combination of LPS and α-MG, respectively. The gene expressions altered after α-MG treatment were involved in pathways related to inflammation-based immune responses, stress responses, regulation of apoptosis and regulation of programmed cell death. Among the approximately 183 genes showing the strongest suppression, 46 genes were related to immune responses and inflammatory responses (Figure 2B). These immune response-related pathways were involved in IL-1 signaling, oncostatin M (OSM) signaling, cytokine production, and Th1 and Th2 cell differentiation.

**Figure 3:**
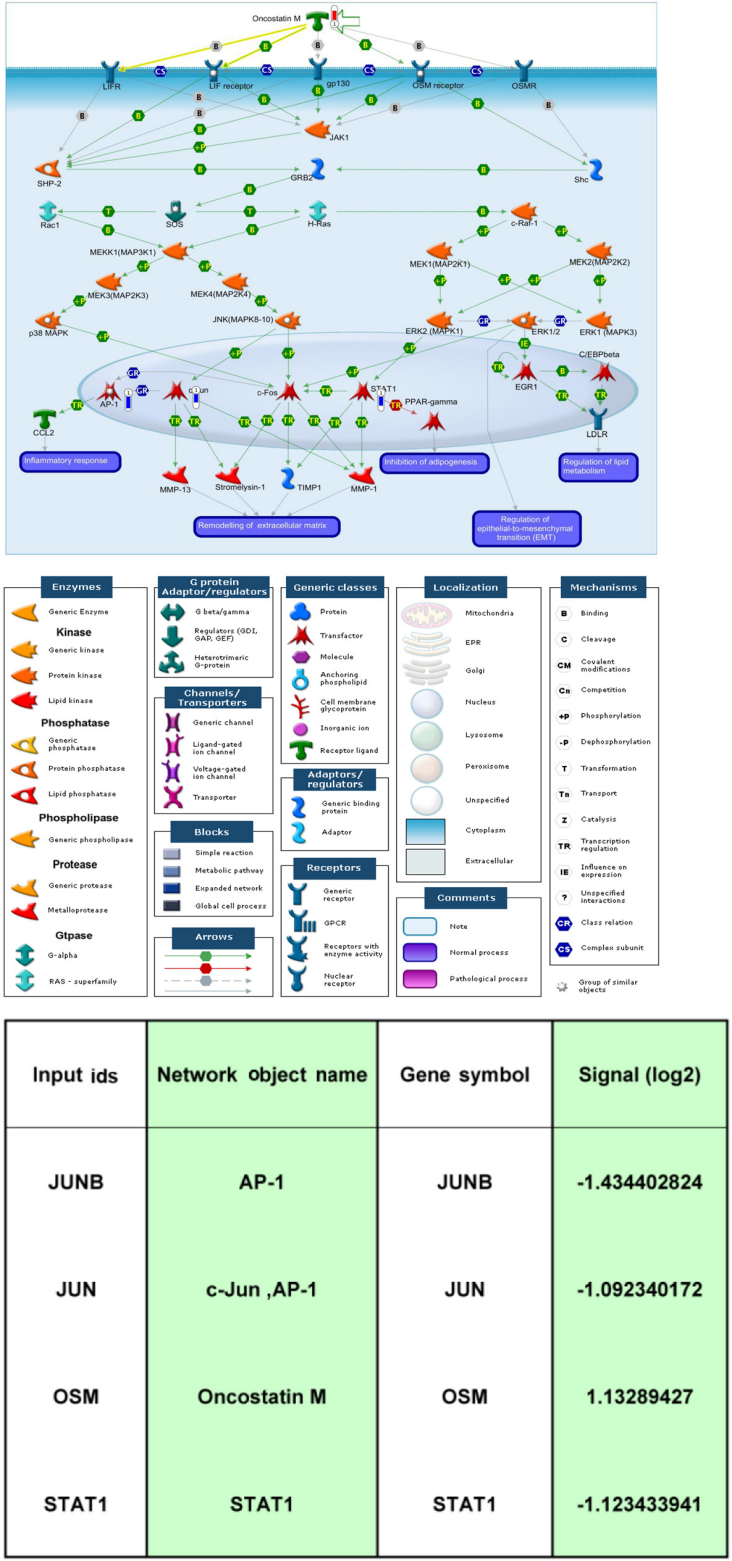
**α-Mangostin regulates OSM signaling in U937 cells.** Four genes (JUNB, JUN, OSM, STAT1) are differential expressed between the LPS-alpha treated state and LPS-only treated state.

OSM is expressed in autoimmune diseases, including rheumatoid arthritis, multiple sclerosis and inflammatory conditions [11]. We observed that α-MG is a promising agent for autoimmune diseases (unpublished data). The results from the microarray showed that JUNB, c-Jun, OSM and STAT1 were differentially expressed between the LPS and α-MG-cotreated and LPS-treated cells in the OSM pathway (Figure 3). α-MG may regulate OSM signaling *via* MAPK pathways and related downstream proteins, including STAT1, c-Jun and c-Fos. The inhibitory actions on three MAPK pathways, ERK1/2, JNK and p38, were examined to delineate the effects of α-MG.

### Decrease in LPS-mediated MAPK activation

**Figure 4:**
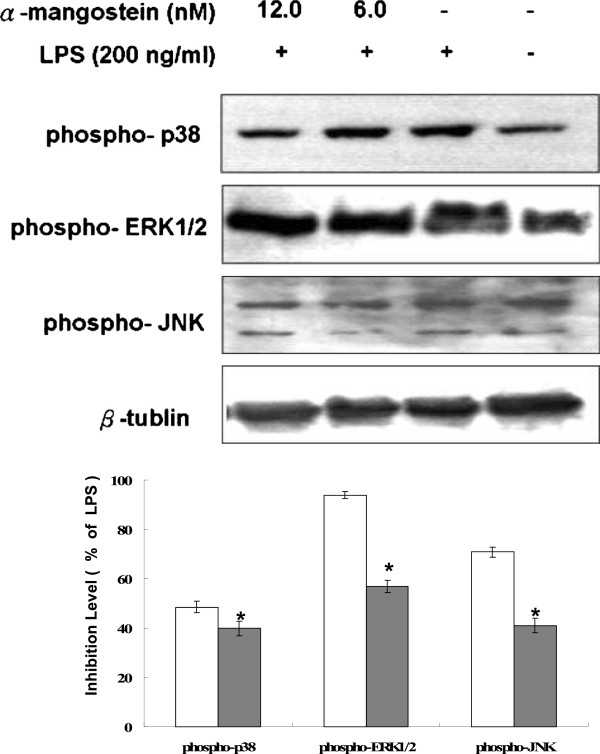
**α-MG decreases LPS-mediated activation of MAPK pathways in U937 cells.** U937 cells were treated with α-MG in the presence of 0.1 ng/mL LPS for 4 h and then lysed. The cell lysates were subjected to Western blotting analyses with ERK1/2, JNK, and p38. Western blots with anti-phospho-ERK1/2, anti-phospho-JNK, and anti-phospho-p38. β-tubulin was evaluated as a loading control, and the protein expression levels were normalized by the corresponding β-tubulin expression levels. Data are expressed as fold phosphorylation normalized to LPS (12 nM α-MG, closed bars; 6 nM α-MG, open bars). All experiments were performed in triplicate and repeated independently three times. *P < 0.05, significant difference from LPS treatment.

LPS treatment induced the phosphorylation of p38, ERK1/2 and JNK, and α-MG treatment attenuated these responses in a dose-dependent manner (*P* = 0.008 for phospho-p38; *P* = 0.016 for phospho-ERK1/2; *P* = 0.01 for phospho-JNK) (Figure 4). The level of p38 phosphorylation was significantly decreased compare with ERK1/2 and JNK (Figure 4). α-MG (12 nM) greatly inhibited p38 phosphorylation, and the phosphorylation was reduced to just 38% of that in LPS-treated cells.

**Figure 5:**
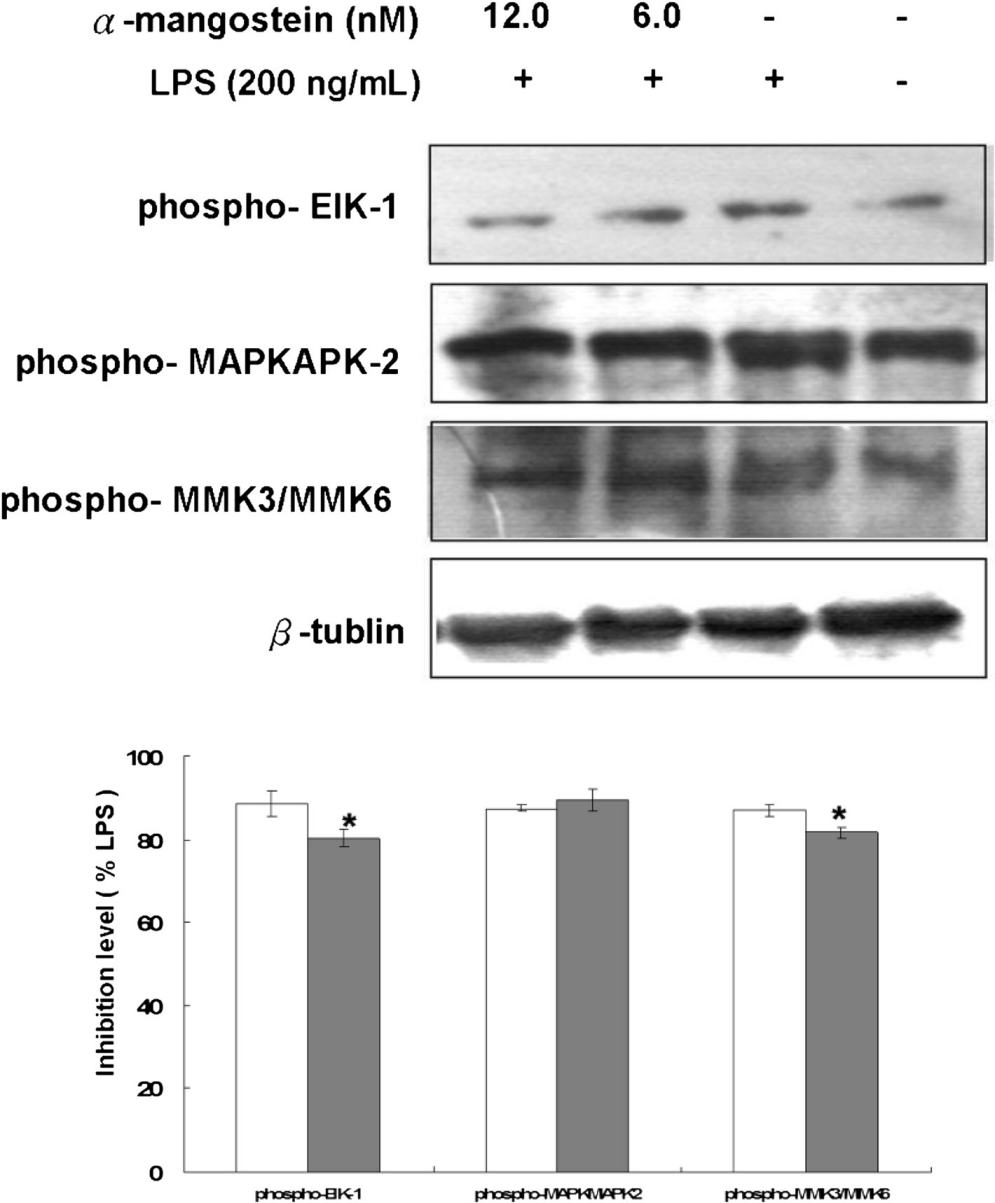
**α-MG decreases LPS-mediated p38 MAPK activation.** U937 cells were treated with α-MG in the presence of 0.1 ng/mL LPS for 4 h and then lysed. The cell lysates were subjected to Western blotting analysis with ELK-1, MMK3/MMK6, and MAPKAPK-2. Western blots with anti-phospho- ELK-1, anti-phospho- MMK3/MMK6, and anti-phospho- MAPKAPK-2. β-tubulin was evaluated as a loading control, and the protein expression levels were normalized by the corresponding β-tubulin expression levels. Data are expressed as fold phosphorylation normalized to LPS (12 nM α-MG, closed bars; 6 nM α-MG, open bars). All experiments were performed in triplicate and repeated independently three times. *P < 0.05, significant difference from LPS treatment.

EIK-1, MMK3/MMK6 and MAPKAPK-2 are substrates of p38 [12], and the effects of α-MG on their phosphorylation were also examined. LPS treatment induced phosphorylation of EIK-1 and MMK3/MMK6, and α-MG treatment attenuated these responses in a concentration-dependent manner (*P* = 0.038 for phospho-EIK-1; *P* = 0.0441 for phospho-MMK3/MMK6; *P* = 0.0453 for phospho- MAPKAPK-2). EIK-1, MMK3/MMK6 and MAPKAPK-2 phosphorylation was greatly inhibited by 12 nM α-MG, and the phosphorylation was reduced to just 78–82% of that in LPS-treated cells (Figure 5). These findings suggest that α-MG exhibits anti-inflammatory activity by inhibiting MAPK phosphorylation, especially in the p38 pathway including EIK-1, MMK3/MMK6 and MAPKAPK-2.

### Regulation of STAT1, c-Jun and c-Fos

**Figure 6:**
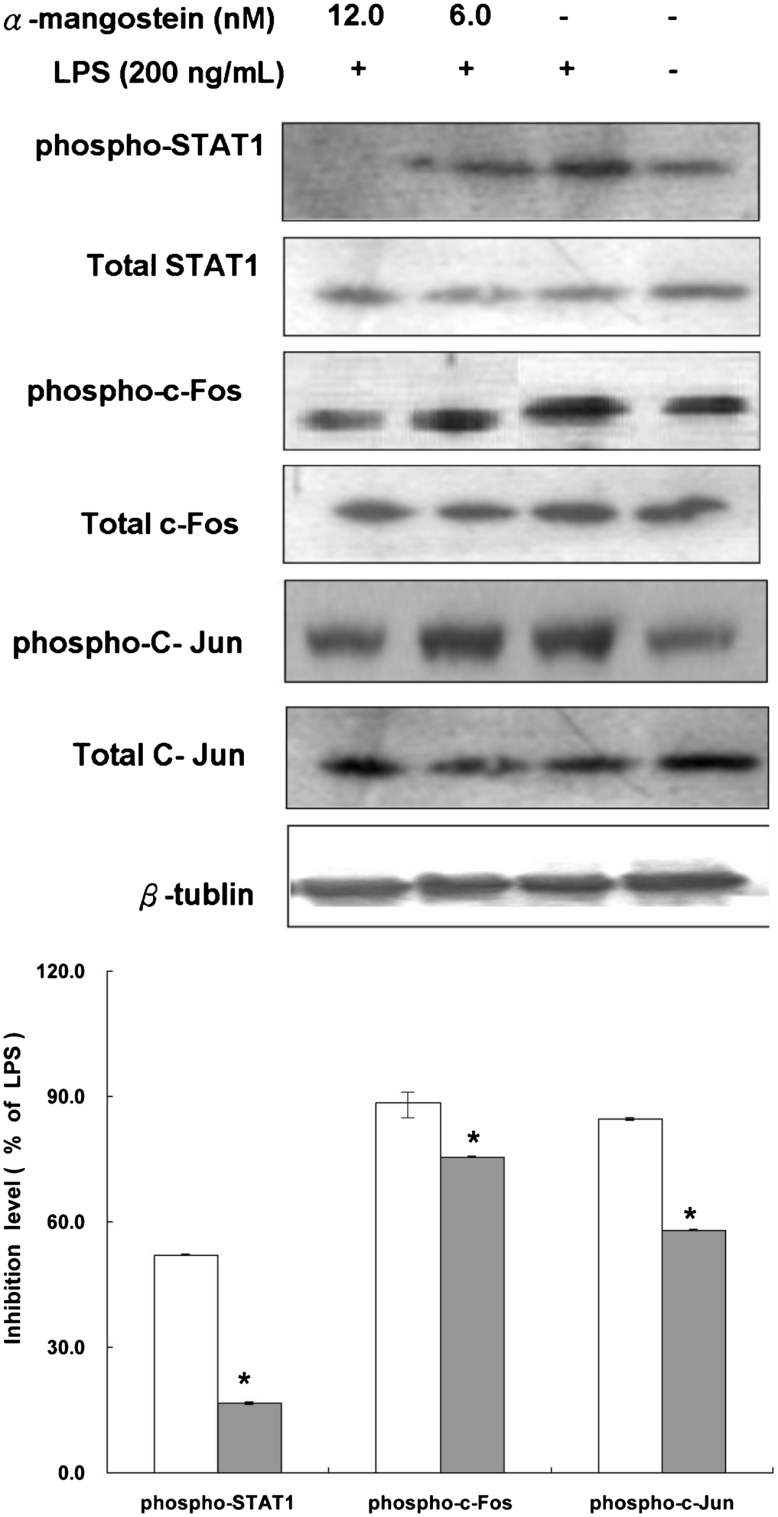
**α-MG regulates STAT 1, c-Jun and c-Fos.** U937 cells were treated with α-MG in the presence of 0.1 ng/mL LPS for 4 h and then lysed. The cell lysates were subjected to Western blotting analyses with STAT 1, c-Jun, c-Fos. Western blots with anti-phospho- STAT 1, anti-phospho- c-Jun, and anti-phospho- c-Fos. β-tubulin was evaluated as a loading control, and the protein expression levels were normalized by the corresponding β-tubulin expression levels. Data are expressed as fold phosphorylation normalized to LPS (12 nM α-MG, closed bars; 6 nM α-MG, open bars). All experiments were performed in triplicate and repeated independently three times. *P < 0.05, significant difference from LPS treatment.

The results from the microarray showed that JUNB, c-Jun, OSM and STAT1 were differentially expressed between the LPS and α-MG-cotreated and LPS-treated cells in the OSM pathway. The protein levels of STAT1, c-Jun and c-Fos were determined by Western blotting analyses. Specifically, α-MG pretreatment attenuated LPS-induced phosphorylation of c-Jun and c-Fos and downstream targets of JNK and ERK1/2 (*P* = 0.04 for phospho-c-Fos) (Figure 6). We demonstrated that α-MG reduced the induction of STAT1 (*P* = 0.0012), c-Jun and c-Fos in a concentration-dependent manner.

**Figure 7:**
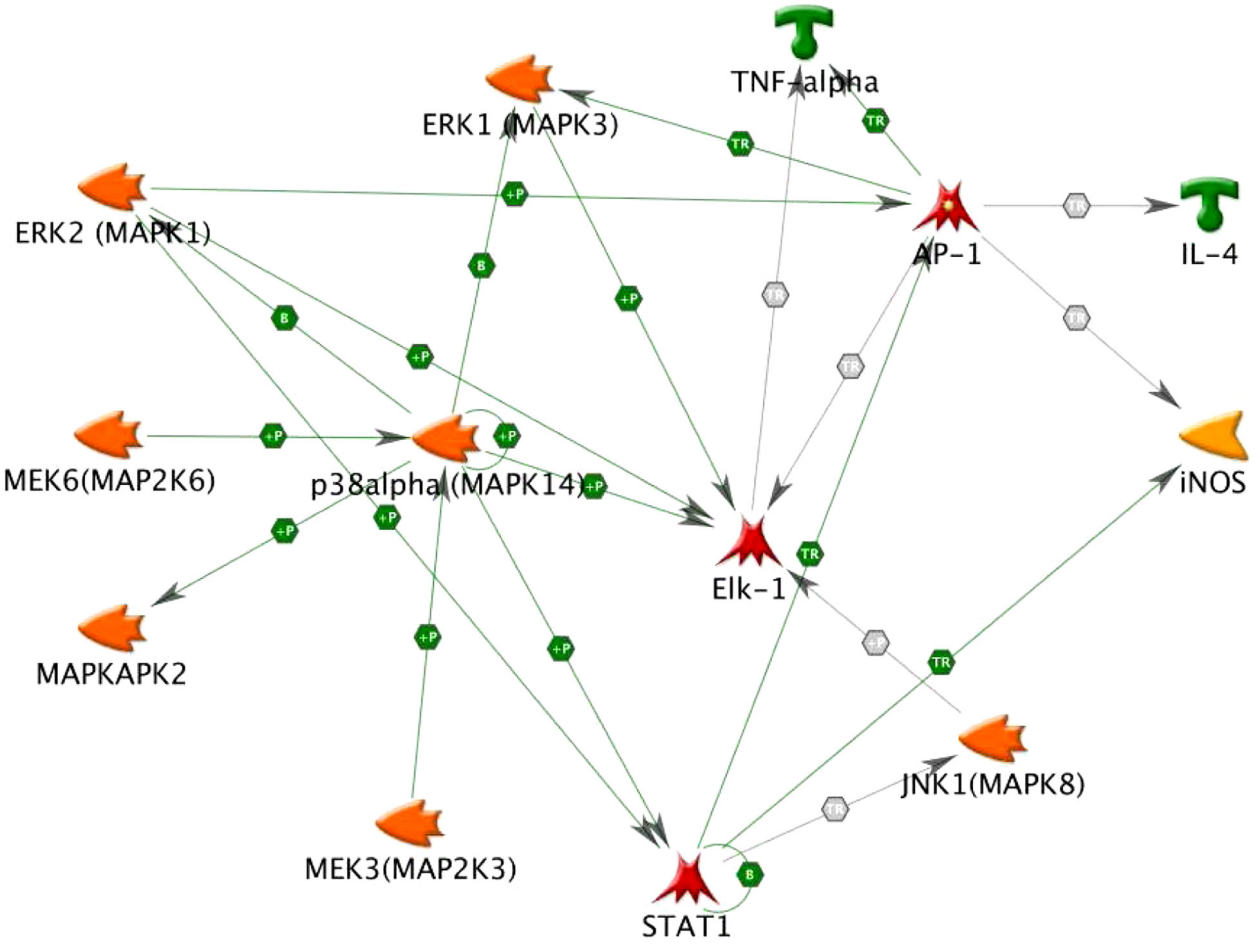
Pathway analysis of α-MG effects on gene expression in U937 cells.

As shown in Figure 7, we have demonstrated that the anti-inflammatory effects of α-MG involves the following: (1) attenuation of LPS-induced production of IL-4 and TNF-α; (2) attenuation of LPS-induced activation of JNK, ERK1/2 and p38; (3) reduction of LPS-induced activation of EIK-1, MMK3/MMK6 and MAPKAPK-2; and (4) attenuation of LPS-mediated suppression of STAT1, c-Jun and c-Fos expression. Taken together, these new findings demonstrate that α-MG inhibits LPS-mediated activation of inflammatory AP-1, MAPK and MAPK-related proteins, including STAT1, c-Jun and c-Fos.

## Conclusion

This study has demonstrated that α-MG attenuates LPS activation of MAPK, STAT1, c-Fos, c-Jun and EIK-1, thereby inhibiting TNF-α and IL-4 production in U937 cells.

## Abbreviations

PMA: Phorbol-12-myristate-13-acetate; IC_50_: Half maximal inhibitory concentration; MTT: 3-(4,5-dimethyl-2-thiazolyl)-2,5-diphenyl-2 H-tetrazolium bromide; PBS: Phosphate-buffered saline; ANOVA: Analysis of variance; AP-1: Activator protein 1; JNK: c-Jun N-terminal kinase; MAPKAPK-2: Mitogen activated protein kinase-activated protein kinase-2; cDNA: Complementary DNA; cRNA: Complementary RNA; ELISA: Enzyme-linked immunosorbent assay; Elk-1: Ets-like molecule 1; ERK1/2: Extracellular signal-regulated kinases 1 and 2; IKK: IκB kinase; IL: Interleukin; iNOS: Inducible NOS; LPS: Lipopolysaccharide; MMK3: MAPK kinase 3; MMK6: MAPK kinase 6; MAPK: Mitogen-activated protein kinase; NF-κB: Nuclear factor-κB; OSM: Oncostatin M; PGE2: Prostaglandin E2; STAT1: Signal transducers and activators of transcription-1.

## Competing interests

The authors declare that they have no competing interests.

## Authors’ contributions

LTL and IM designed the study. SHL performed the Western blotting and statistical analyses, and wrote the manuscript. NYH, KKH and YCS performed the cytokines assay experiments. JML, TYC, WHW and TSC performed the microarray experiments and wrote the manuscript. All authors read and approved the final manuscript.
